# Environmental effects of COVID-19 pandemic and potential strategies of sustainability

**DOI:** 10.1016/j.heliyon.2020.e04965

**Published:** 2020-09-17

**Authors:** Tanjena Rume, S.M. Didar-Ul Islam

**Affiliations:** a Department of Geological Sciences, Jahangirnagar University, Dhaka 1342, Bangladesh; b Department of Environmental Sciences, Jahangirnagar University, Dhaka 1342, Bangladesh

**Keywords:** Environmental assessment, Environmental pollution, Environmental management, Environmental sustainability, COVID-19, Public health, Lockdown, GHGs emission, Biomedical waste

## Abstract

The global outbreak of coronavirus disease 2019 (COVID-19) is affecting every part of human lives, including the physical world. The measures taken to control the spread of the virus and the slowdown of economic activities have significant effects on the environment. Therefore, this study intends to explore the positive and negative environmental impacts of the COVID-19 pandemic, by reviewing the available scientific literatures. This study indicates that, the pandemic situation significantly improves air quality in different cities across the world, reduces GHGs emission, lessens water pollution and noise, and reduces the pressure on the tourist destinations, which may assist with the restoration of the ecological system. In addition, there are also some negative consequences of COVID-19, such as increase of medical waste, haphazard use and disposal of disinfectants, mask, and gloves; and burden of untreated wastes continuously endangering the environment. It seems that, economic activities will return soon after the pandemic, and the situation might change. Hence, this study also outlines possible ways to achieve long-term environmental benefits. It is expected that the proper implementation of the proposed strategies might be helpful for the global environmental sustainability.

## 1 Introduction

**Figure 1:**
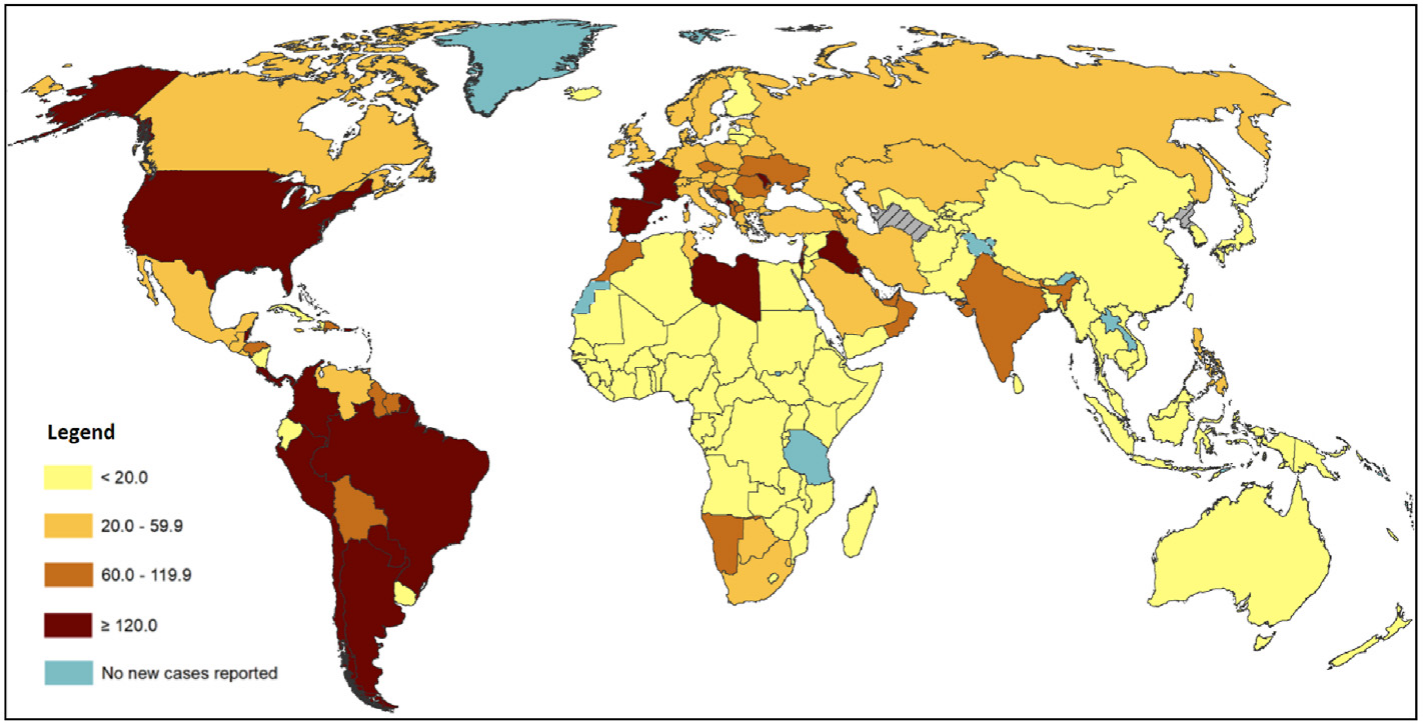
Geographic distribution of 14-day cumulative number of reported COVID-19 cases per 100000 populations, as of September 06, 2020 (Source: ECDC, 2020).

**Figure 2:**
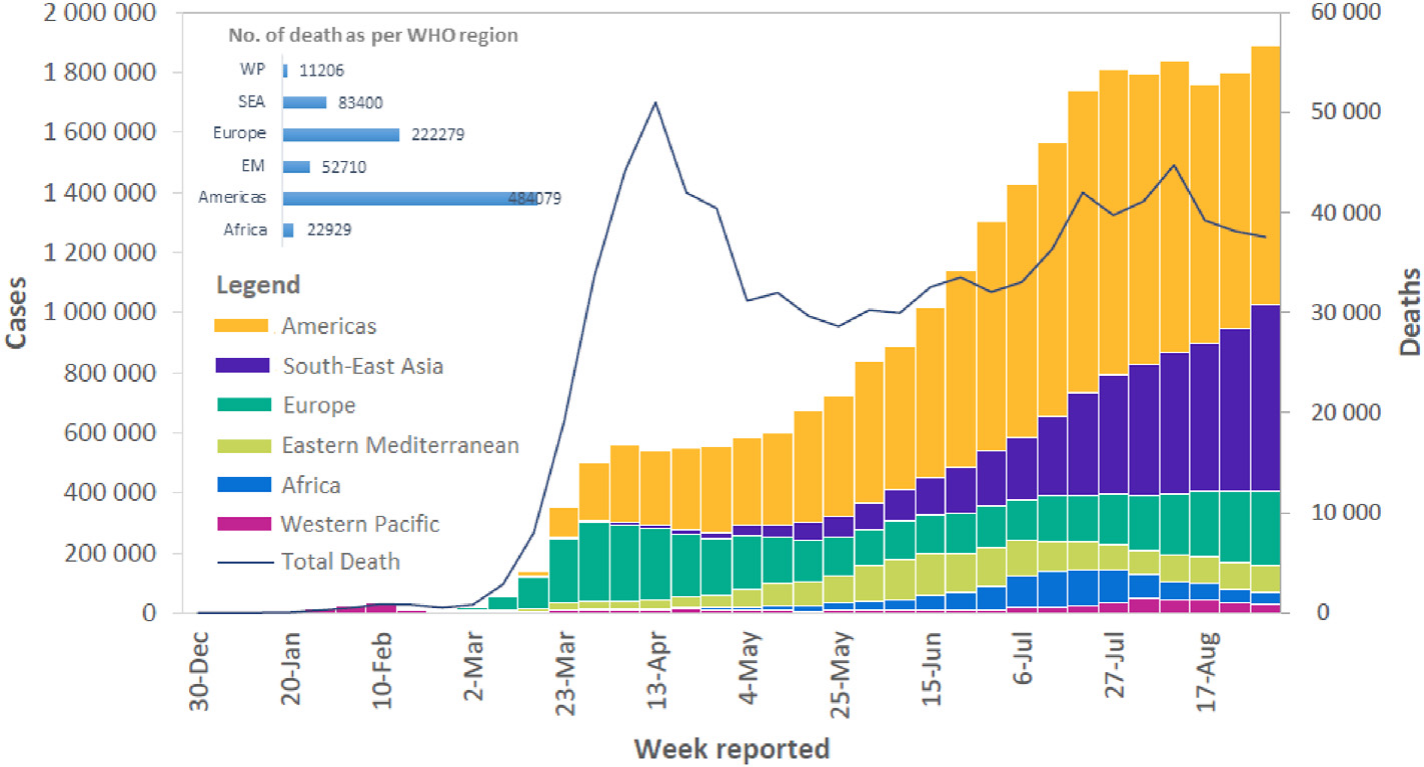
Number of COVID-19 cases reported weekly by WHO region, and total deaths, up to September 06, 2020 (Data source: WHO, 2020c).

The outbreak of coronavirus disease-2019 (COVID-19) first emerged at the end of December 2019, from the Hunan seafood market in Wuhan City of China, and declared as an international public health emergency in a couple of weeks by the World Health Organization (WHO, 2020a). It is an infectious disease caused by severe acute respiratory syndrome coronavirus-2 (SARS-CoV-2) (Islam et al., 2020; Nghiem et al., 2020; Wang et al., 2020). Genomic analysis revealed that SARS-CoV-2 is phylogenetically associated with SARS viruses, and bats could be the possible primary source (Chakraborty and Maity, 2020). Although the intermediate source of origin and transfer to humans is not clearly known, the rapid human to human transmission capability of this virus has been established (Hui et al., 2020). The transmission of the virus mainly occurred through person-to-person via direct contact or droplets produced by coughing, sneezing and talking (Islam et al., 2020; Li et al., 2020; Wang et al., 2020). As of September 06, 2020; the virus has claimed to spread 216 countries, areas or territories with the death of 876, 616 humans from 26,763,217 confirmed cases (WHO, 2020a), and the number is increasing rapidly. The geographic distribution of COVID-19 cases (Figure 1), and the epidemic curve indicating the number of confirmed cases and deaths in different parts of the world are illustrated in Figure 2.

**Figure 3:**
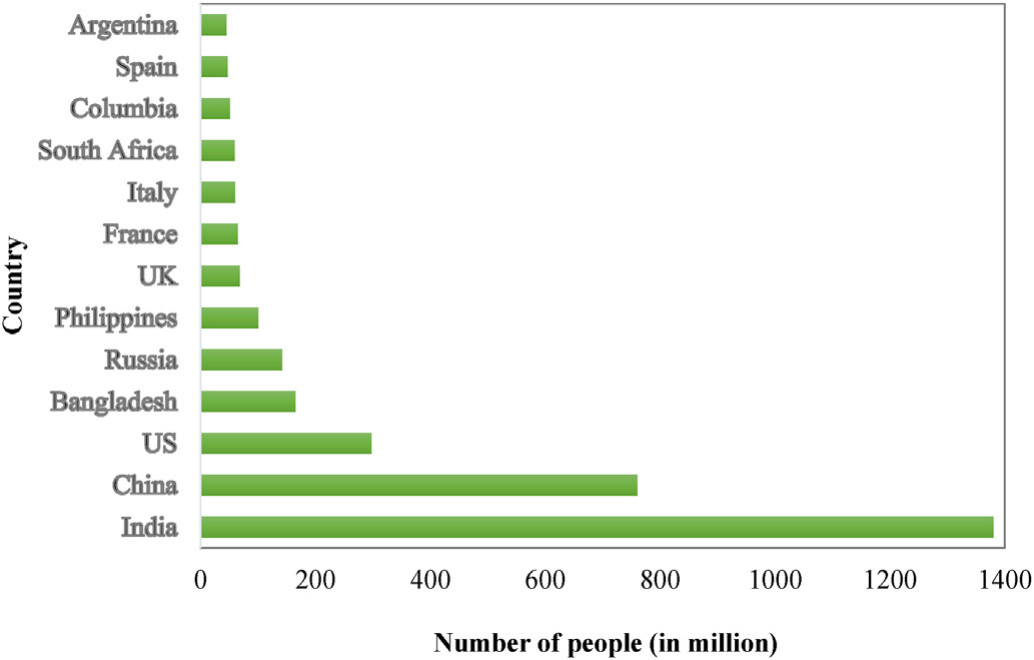
Global example of the number of people (as of April 23, 2020) placed on enforced lockdown during the outbreak of COVID-19 (Data source: Buchholz, 2020).

Usually, the symptoms of COVID-19 infection include fever, chills, cough, sore throat, breathing difficulty, myalgia or fatigue, nausea, vomiting, and diarrhoea (Huang et al., 2020; Wang et al., 2020). Severe cases can lead to cardiac injury, respiratory failure, acute respiratory distress syndrome, and even death (Holshue, 2020; Wang et al., 2020). Older people along with other underlying medical conditions are at a high risk of mortality (Chen, 2020). Till date, there has not been any significant breakthrough in the development of an effective medicine or a vaccine for this disease. National and international authorities and experts suggest the use of non-pharmaceutical measures like wearing face masks and hand gloves, washing hands with soap, frequent use of antiseptic solution and maintaining social distance (Hui et al., 2020; Sajed and Amgain, 2020; WHO, 2020b). To control the spread of the virus and reduce the death rate, government of most of the affected countries initiated to restrict the movement of people. Figure 3 illustrates global examples of the country wise number of people placed on enforced lockdown due to the coronavirus pandemic. It is found that India restricted the movement of the largest number of people (approximately 1.3 billion) as a preventive measure of COVID-19, which started from March 24, 2020 (Somani et al., 2020). Except emergency services (e.g., medical, fire, police, food supply etc.), all other organizations including educational institutions are being closed to encourage people to stay at home. All the public transport services (e.g., bus, truck, train, aeroplanes etc.) were suspended, with exceptions of the transportation of essential goods and emergency services (Tripathi, 2020). In Italy, the most extensive travel restrictions are placed after the second World War (Cellini et al., 2020). In London, the typically bustling pubs, bars and theatres have been closed, and people have been advised to stay at home. As of April 7, 2020, World Economic Forum reported, nearly 3 billion people are faced with some form of lockdown globally, and movement is being restricted by respective governments to control the COVID-19 infection (WEF, 2020). Overall, the pandemic has caused huge global socio-economic disruption, which directly or indirectly affected the environment like improvement of air and water quality, reduction of noise and restoration of ecology (Chakraborty and Maity, 2020; Somani et al., 2020; Saadat et al., 2020). Moreover, the increased use of personal protective equipment (PPE) (e.g., face mask, hand gloves, gowns, goggles, face shield etc.), and their haphazard disposal creates environmental burden (Fadare and Okoffo, 2020; Nghiem et al., 2020; Singh et al., 2020). In these circumstances, this study intended to explore the positive and negative environmental consequences of the COVID-19 pandemic, and propose possible strategies as future guideline for environmental sustainability.

## 2 Methodology

This study was performed by reviewing the available published literatures, case studies, and different government and non-government organizations information from reports and official websites. Scientific literatures were collected through electronic means from the database of Science Direct, Springer, PubMed, Tailor and Francis, ISI Web of Knowledge, Research Gate, and Google Scholar but not in a systematic manner. From a large number of studies, this study compiles and presents the data and information which are relevant to the environmental effects of COVID-19 and meet the study goals.

## 3 Environmental effects of COVID-19

**Figure 4:**
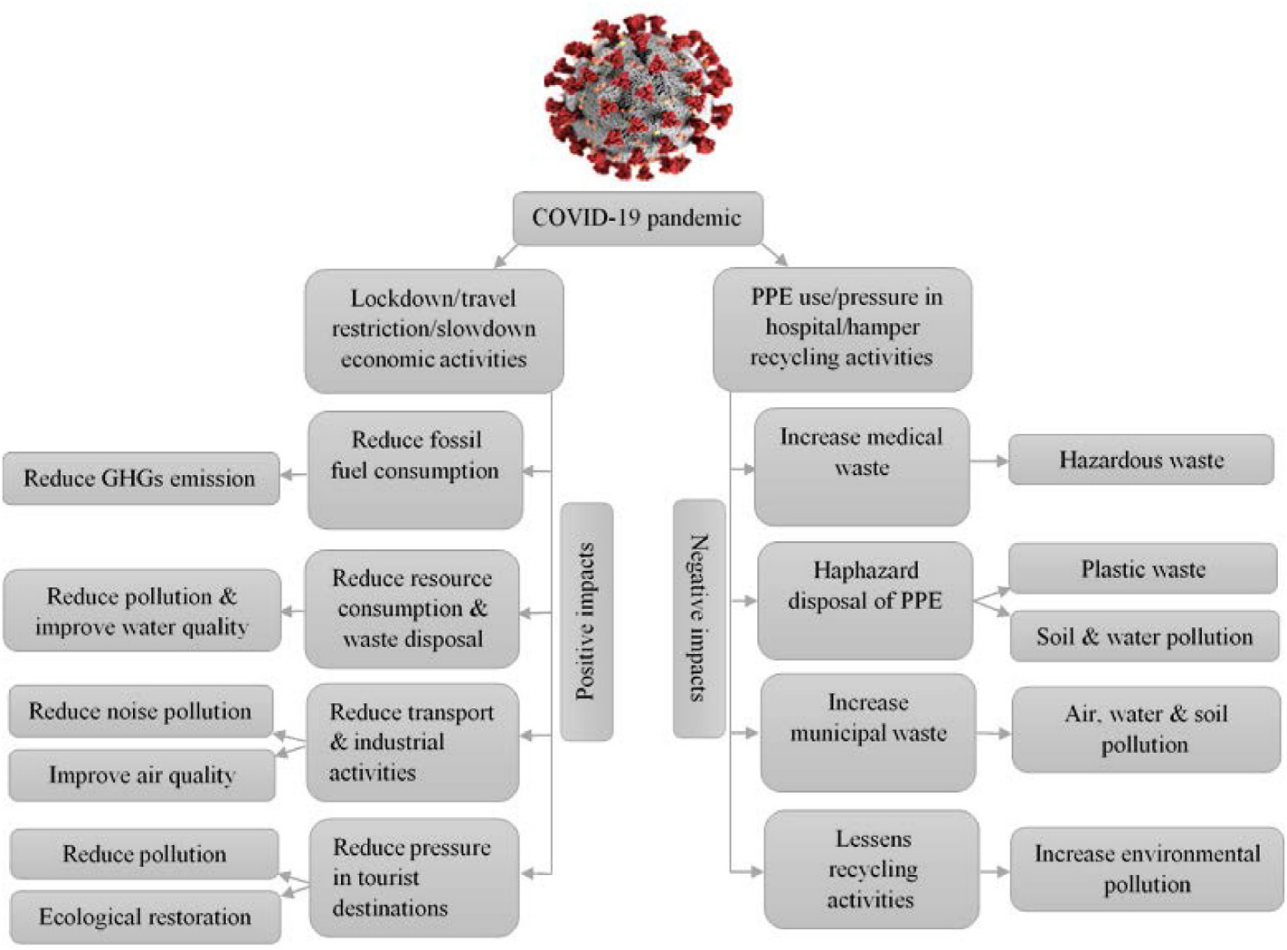
Positive and negative environmental effects of COVID-19 pandemic.

The global disruption caused by the COVID-19 has brought about several effects on the environment and climate. Due to movement restriction and a significant slowdown of social and economic activities, air quality has improved in many cities with a reduction in water pollution in different parts of the world. Besides, increased use of PPE (e.g., face mask, hand gloves etc.), their haphazard disposal, and generation of a huge amount of hospital waste has negative impacts on the environment. Both positive and negative environmental impacts of COVID-19 are present in Figure 4.

### 3.1 Positive environmental effects

#### 3.1.1 Reduction of air pollution and GHGs emission

As industries, transportation and companies have closed down, it has brought a sudden drop of greenhouse gases (GHGs) emissions. Compared with this time of last year, levels of air pollution in Ney York has reduced by nearly 50% because of measures taken to control the virus (Henriques, 2020). It was estimated that nearly 50% reduction of N_2_O and CO occurred due to the shutdown of heavy industries in China (Caine, 2020). Also, emission of NO₂ is one of the key indicators of global economic activities, which indicates a sign of reduction in many countries (e.g., US, Canada, China, India, Italy, Brazil etc.) due to the recent shut down (Biswal et al., 2020; Ghosh, 2020; Saadat et al., 2020; Somani et al., 2020). Usually, NO_2_ is emitted from the burning of fossil fuels, 80% of which comes from motor vehicle exhaust (USEPA, 2016). It is reported that NO_2_ causes acid rain with the interaction of O_2_ and H_2_O, and several respiratory diseases suffered by humans (USEPA, 2016). The European Environmental Agency (EEA) predicted that, because of the COVID-19 lockdown, NO_2_ emission dropped from 30-60% in many European cities including Barcelona, Madrid, Milan, Rome and Paris (EEA, 2020). In the US NO_2_ declined 25.5% during the COVID-19 period compared to previous years (Berman and Edisu, 2020). The level of NO_2_ demonstrated a reduction across Ontario (Canada) and found to be reduced from 4.5 ppb to 1 ppb (Adams, 2020). Up to 54.3% decrease of NO_2_ was observed in Sao Paulo of Brazil (Nakada and Urban, 2020). It was also stated that, the levels of NO_2_ and PM_2.5_ reduced by almost 70% in Delhi, the capital of India (Thiessen, 2020). Overall, 46% and 50% reduction of PM2.5 and PM_10_ respectively, was reported in India during the nationwide lockdown (IEP, 2020).

It is assumed that, vehicles and aviation are key contributors of emissions and contribute almost 72% and 11% of the transport sector's GHGs emission respectively (Henriques, 2020). The measures taken globally for the containment of the virus are also having a dramatic impact on the aviation sector. Many countries restricted international travelers from entry and departure. Due to the decreased passengers and restrictions, worldwide flights are being cancelled by commercial aircraft companies. For instance, China reduces almost 50–90% capacity of departing and 70% domestic flights due to the pandemic, compared to January 20, 2020, which ultimately deducted nearly 17% of national CO_2_ emissions (Zogopoulos, 2020). Furthermore, it is reported that 96% of air travel dropped from a similar time last year globally due to the COVID-19 pandemic (Wallace, 2020), which has ultimate effects on the environment.

**Figure 5:**
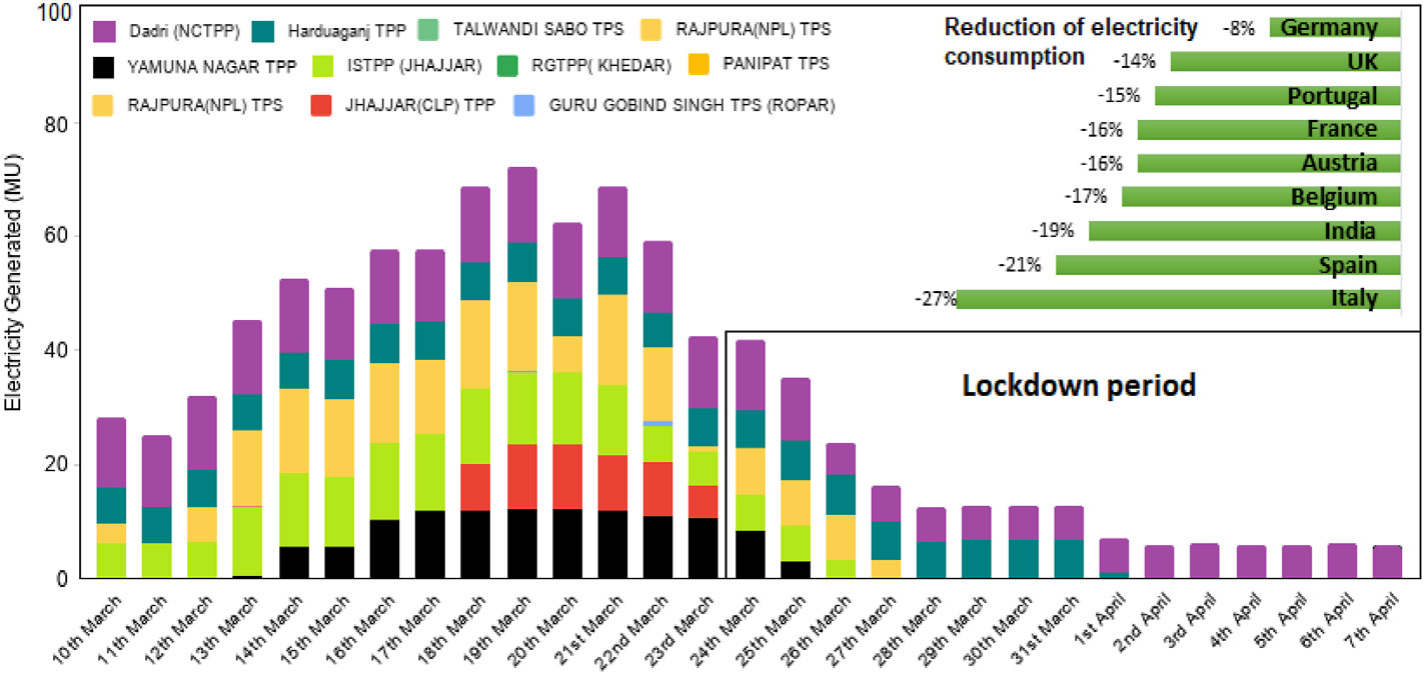
Coal based electricity generation scenario before and after lockdown in the periphery of Delhi, India, along with total electricity consumption reduction in some selected countries (Data sources: Armstrong, 2020; CREA, 2020).

Overall, much less consumption of fossil fuels lessens the GHGs emission, which helps to combat against global climate change. According to the International Energy Agency (IEA), oil demand has dropped 435,000 barrels globally in the first three months of 2020, compared to the same period of last year (IEA, 2020). Besides, global coal consumption is also reduced because of less energy demand during the lockdown period (Figure 5). It is reported that, coal-based power generation reduced 26% in India with 19% reduction of total power generation after lockdown (CREA, 2020). Again, China, the highest coal consumer in the world, dropped 36% compared to same time of the preceding year (early February to mid-march) (CREA, 2020; Ghosh, 2020). According to UK based climate science and policy website Carbon Brief, recent crisis of COVID-19 reduces 25% CO_2_ emission in China, and nonetheless below the normal limit more than two months after the country entered lockdown (Evans, 2020). They also projected that, the pandemic could cut 1,600 metric tons of CO_2_, equivalent to above 4% of the global total in 2019 (Evans, 2020).

#### 3.1.2 Reduction of water pollution

Water pollution is a common phenomenon of a developing country like India, and Bangladesh, where domestic and industrial wastes are dumped into rivers without treatment (Islam and Azam, 2015; Islam and Huda, 2016; Bodrud-Doza et al., 2020; Yunus et al., 2020). During the lockdown period, the major industrial sources of pollution have shrunk or completely stopped, which helped to reduce the pollution load (Yunus et al., 2020). For instance, the river Ganga and Yamuna have reached a significant level of purity due to the absence of industrial pollution on the days of lockdown in India. It is found that, among the 36 real-time monitoring stations of river Ganga, water from 27 stations met the permissible limit (Singhal and Matto, 2020). This improvement of water quality at Haridwar and Rishikesh was ascribed to the sudden drop of the number of visitors and 500% reduction of sewage and industrial effluents (Singhal and Matto, 2020; Somani et al., 2020). According to the real-time water quality monitoring data of the Uttarakhand Pollution Control Board (UPCB, 2020) of India, physicochemical parameters i.e, pH (7.4–7.8), dissolved oxygen (DO) (9.4–10.6 mg/L), biochemical oxygen demand (BOD) (0.6–1.2 mg/L) and total coliform (40–90 MPN/100 mL) of the river Ganga was found within the surface water quality standard of India. Except total coliform in some monitoring stations, all others parameters even meet the national drinking water quality standard, which can be used without conventional treatment but after disinfection (Class A) (BIS, 2012). It is also found that, the concentration of pH, electric conductivity (EC), DO, BOD and chemical oxygen demand (COD) has reduced almost 1–10%, 33–66%, 45–90%, and 33–82% respectively in different monitoring stations during the lockdown in comparison to the pre-lockdown period (Arif et al., 2020). Moreover, due to imposed a ban of public gathering, number of tourists and water activities were reduced in many places (Cripps, 2020; Zambrano-Monserrate et al., 2020). It is reported that, due to the lockdown of COVID-19, the Grand Canal of Italy turned clear, and reappearances of many aquatic species (Clifford, 2020). Water pollution are also reduced in the beach areas of Bangladesh, Malaysia, Thailand, Maldives, and Indonesia (Kundu, 2020; Rahman, 2020). Jribi et al. (2020) reported that, due to the COVID-19 lockdown, the amount of food waste is reduced in Tunisia, which ultimately reduces soil and water pollution. However, the amount of industrial water consumption is also reduced, especially from the textile sector around the glove (Cooper, 2020). Usually, huge amount of solid trashes is generated from construction and manufacturing process responsible for water and soil pollution, also reduced. Moreover, owing to the reduction of export-import business, the movement of merchant ship and other vessels are reduced globally, which also reduces emission as well as marine pollution.

#### 3.1.3 Reduction of noise pollution

Noise pollution is the elevated levels of sound, generated from different human activities (e.g., machines, vehicles, construction work), which may lead to adverse effects in human and other living organisms (Goines and Hagler, 2007; Zambrano-Monserrate et al., 2020). Usually, noise negatively effects on physiological health, along with cardiovascular disorders, hypertension, and sleep shortness of human (Kerns et al., 2018). It is reported that, globally around 360 million people are prone to hearing loss due to noise pollution (Sims, 2020). World Health Organization predicted that in Europe alone, over 100 million people are exposed to high noise levels, above the recommended limit (WHO, 2012). Moreover, anthropogenic noise pollution has adverse impacts on wildlife through the changing balance in predator and prey detection and avoidance. Unwanted noise also negatively effects on the invertebrates, that help to control environmental processes which are vital for the balance of the ecosystem (Solan et al., 2016). However, the quarantine and lockdown measures mandate that people stay at home and reduced economic activities and communication worldwide, which ultimately reduced noise level in most cities (Zambrano-Monserrate et al., 2020). For instance, noise level of Delhi the capital of India, is reduced drastically around 40–50% in the recent lockdown period (Somani et al., 2020). Due to reduction of vehicle movement during the lockdown period, the noise levels of Govindpuri metro station (Delhi) is reduced 50–60 dB, from 100 dB (Gandhiok and Ibra, 2020). According to the Central Pollution Control Board (CPCB, 2020) of India, noise level of residential area of Delhi is reduced 55 dB (daytime) and 45 dB (night) to 40 dB (daytime) and 30 dB (night) respectively. As a result, city dwellers are now enjoying the chirping of birds, which usually ranges from 40-50 dB (Gandhiok and Ibra, 2020). Moreover, due to travel restrictions, the number of flights and vehicular movements have drastically reduced around the world, which have ultimately reduced the level of noise pollution. For example, in Germany passenger air travel has been slashed by over 90%, car traffic has dropped by >50% and trains are running <25% than the usual rates (Sims, 2020). Overall, COVID-19 lockdown, and lessens of economic activities reduced the noise pollution around the globe.

#### 3.1.4 Ecological restoration and assimilation of tourist spots

Over the past few years, tourism sector has witnessed a remarkable growth because of technological advancements and transport networks; which contribute significantly to global gross domestic product (GDP) (Lenzen et al., 2018). It is estimated that the tourism industry is responsible for 8% of global GHGs emission (Lenzen et al., 2018). However, the places of natural beauty (e.g., beaches, islands, national park, mountains, desert and mangroves) are usually attracting the tourists, and make a huge harsh. To facilitate and accommodate them, lots of hotels, motel, restaurant, bar and market are built, which consume lots of energy and other natural resources (Pereira et al., 2017). For instance, Puig et al. (2017) calculated the carbon footprint of coastland hotel services of Spain and reported electricity and fuels consumption take a key role, and 2-star hotels have the highest carbon emissions. Moreover, visitors dump various wastes which impair natural beauty and create ecological imbalance (Islam and Bhuiyan, 2018). Due to the outbreak of COVID-19 and local restrictions, the number of tourists have reduced in the tourist spots around the world (Zambrano-Monserrate et al., 2020). For instance, Phuket, Thailand's most popular tourist's destination goes into lockdown on April 9, 2020, due to the surge of Covid-19, where an average 5,452 visitors visit per day (Cripps, 2020). Similarly, local administration imposed a ban on public gathering and tourist arrivals at Cox's Bazar sea beach, known as the longest unbroken natural sand sea beach in the world. As a result of restriction, the color of sea water is changed, which usually remain turbid because of swimming, bathing, playing and riding motorized boats (Rahman, 2020). Nature gets a time to assimilate human annoyance, and due to pollution reduction recently returning of dolphins was reported in the coast of Bay of Bengal (Bangladesh) and canals, waterways, and ports of Venice (Italy) after a long decade (Rahman, 2020; Kundu, 2020).

### 3.2 Negative environmental effects

#### 3.2.1 Increase of biomedical waste generation

Since the outbreak of COVID-19, medical waste generation is increased globally, which is a major threat to public health and environment. For sample collection of the suspected COVID-19 patients, diagnosis, treatment of huge number of patients, and disinfection purpose lots of infectious and biomedical wastes are generated from hospitals (Somani et al., 2020; Zambrano-Monserrate et al., 2020). For instance, Wuhan in China produced more than 240 metric tons of medical wastes every day during the time of the outbreak (Saadat et al., 2020), which is almost 190 m tonnes higher than the normal time (Zambrano-Monserrate et al., 2020). Again, in the city of Ahmedabad of India, the amount of medical waste generation is increased from 550-600 kg/day to around 1000 kg/day at the time of the first phase of lockdown (Somani et al., 2020). Around 206 m tonnes of medical waste are generated per day in Dhaka, the capital of Bangladesh because of COVID-19 (Rahman et al., 2020). Also other cities like Manila, Kuala Lumpur, Hanoi, and Bangkok experienced similar increases, producing 154–280 m tonnes more medical waste per day than before the pandemic (ADB, 2020). Such a sudden rise of hazardous waste, and their proper management has become a significant challenge to the local waste management authorities. According to the recent published literature, it is reported that the SARS-CoV-2 virus can exist a day on cardboard, and up to 3 days on plastics and stainless steel (Van-Doremalen et al., 2020). So, waste generated from the hospitals (e.g., needles, syringes, bandage, mask, gloves, used tissue, and discarded medicines etc.) should be managed properly, to reduce further infection and environmental pollution, which is now a matter of concern globally.

#### 3.2.2 Safety equipment use and haphazard disposal

To protect from the viral infection, presently peoples are using face mask, hand gloves and other safety equipment, which increase the amount of healthcare waste. It is reported that, in USA, trash amount has been increasing due to increased PPE use at the domestic level (Calma, 2020). Since the outbreak of COVID-19, the production and use of plastic based PPE is increased worldwide (Singh et al., 2020). For instance, China increased the daily production of medical masks to 14.8 million since from February 2020, which is much higher than before (Fadare and Okoffo, 2020). However, due to lack of knowledge about infectious waste management, most people dump these (e.g., face mask, hand gloves etc.) in open places and in some cases with household wastes (Rahman et al., 2020). Such haphazard dumping of these trashes creates clogging in water ways and worsens environmental pollution (Singh et al., 2020; Zambrano-Monserrate et al., 2020). It is reported that, face mask and other plastic based protective equipment are the potential source of microplastic fibers in the environment (Fadare and Okoffo, 2020). Usually, Polypropylene is used to make N-95 masks, and Tyvek for protective suits, gloves, and medical face shields, which can persist for a long time and release dioxin and toxic elements to the environment (Singh et al., 2020). Though, experts and responsible authorities suggest for the proper disposal and segregation of household organic waste and plastic based protective equipment (hazardous medical waste), but mixing up these wastes increases the risk of disease transmission, and exposure to the virus of waste workers (Ma et al., 2020; Somani et al., 2020; Singh et al., 2020).

#### 3.2.3 Municipal solid waste generation, and reduction of recycling

Increase of municipal waste (both organic and inorganic) generation has direct and indirect effects on environment like air, water and soil pollution (Islam et al., 2016). Due to the pandemic, quarantine policies established in many countries have led to an increase in the demand of online shopping for home delivery, which ultimately increase the amount of household wastes from shipped package materials (Somani et al., 2020; Zambrano-Monserrate et al., 2020). However, waste recycling is an effective way to prevent pollution, save energy, and conserve natural resources (Ma et al., 2019). But, due to the pandemic many countries postponed the waste recycling activities to reduce the transmission of viral infection. For instance, USA restricted recycling programs in many cities (nearly 46%), as government worried about the risk of COVID-19 spreading in recycling facilities (Somani et al., 2020). United Kingdom, Italy, and other European countries also prohibited infected residents from sorting their waste (Zambrano-Monserrate et al., 2020). Overall, due to disruption of routine municipal waste management, waste recovery and recycling activities, increasing the landfilling and environmental pollutants worldwide.

#### 3.2.4 Other effects on the environment

Recently, huge amount of disinfectants is applied into roads, commercial, and residential areas to exterminate SARS-CoV-2 virus. Such extensive use of disinfectants may kill non-targeted beneficial species, which may create ecological imbalance (Islam and Bhuiyan, 2016). Moreover, SARS-CoV-2 virus was detected in the COVID-19 patient's faeces and also from municipal wastewater in many countries including Australia, India, Sweden, Netherlands and USA (Ahmed et al., 2020; Nghiem et al., 2020; Mallapaty, 2020). So, additional measures in wastewater treatment are essential, which is challenging for developing countries like Bangladesh, where municipal wastewater is drained into nearby aquatic bodies and rivers without treatment (Islam and Azam, 2015; Rahman and Islam, 2016). China has already strengthened the disinfection process (increased use of chlorine) to prevent SARS-CoV-2 virus spreading through the wastewater. But, the excessive use of chlorine in water could generate harmful by-product (Zambrano-Monserrate et al., 2020).

## 4 Potential strategies of environmental sustainability

**Figure 6:**
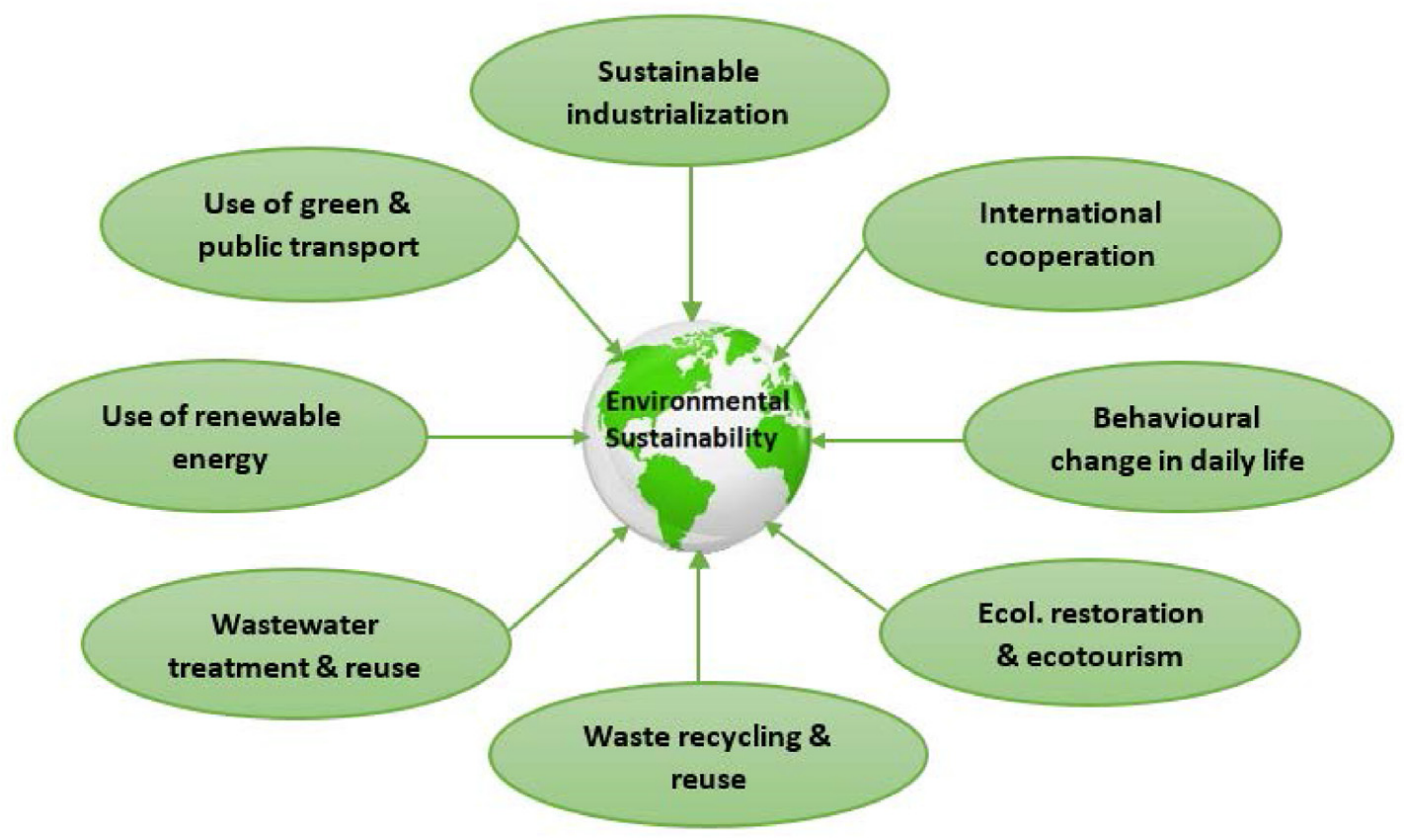
Proposed strategies of sustainable environmental management.

It is assumed that, all of these environmental consequences are short-term. So, it is high time to make a proper strategy for long-term benefit, as well as sustainable environmental management. The COVID-19 pandemic has elicited a global response and make us united to win against the virus. Similarly, to protect this globe, the home of human beings, united effort of the countries should be imperative (Somani et al., 2020). Therefore, some possible strategies are proposed for global environmental sustainability (Figure 6).

i ***Sustainable industrialization:*** Industrialization is crucial for economic growth; however, it's time to think about sustainability. For sustainable industrialization, it is essential to shift to less energy-intensive industries, use of cleaner fuels and technologies, and strong energy efficient policies (Pan, 2016). Moreover, industries should be built in some specific zones, keeping in mind that waste from one industry can be used as raw materials of the other (Hysa et al., 2020). After a certain period, industrial zones should have been shut down in a circular way to reduce emission without hampering the national economy. Again, industries especially readymade garments (RMG) and others where a huge number of people work, proper distance and hygienic environment should maintain to reduce the spread of any infectious communicable disease.
ii ***Use of green and public transport:*** To reduce emissions, it is necessary to encourage people to use public transport, rather private vehicles. Besides, people should encourage to use bicycle in a short distance, and public bike sharing (PBS) system (like China) should be available for mass usage, which is not only environment friendly but also beneficial for health.
iii ***Use of renewable energy:*** Use of renewable energy can lower the demand of fossil fuels like coal, oil, and natural gas, which can play an important role in reducing the GHGs emissions (Ellabban et al., 2014; CCAC, 2019). Due to the COVID-19 pandemic, global energy demand is reduced, which results in the reduction of emission and increased ambient air quality in many areas (Somani et al., 2020; Zambrano-Monserrate et al., 2020). But, to maintain the daily needs and global economic growth, it is not possible to cut-off energy demand like a pandemic situation. Hence, use of renewable energy sources like solar, wind, hydropower, geothermal heat and biomass can meet the energy demand and reduces the GHGs emission (Ellabban et al., 2014).
iv ***Wastewater treatment and reuse:*** To control the challenges of water pollution, both industrial and municipal wastewater should be properly treated before discharge. Besides, reuse of treated wastewater in non-production processes like toilet flushing and road cleaning can reduce the burden of excess water withdrawal.
v ***Waste recycling and reuse:*** To reduce the burden of wastes and environmental pollution, both industrial and municipal wastes should be recycled and reused. Hence, circular economy or circularity systems should implement in the production process to minimize the use of raw material and waste generation (Hysa et al., 2020). Moreover, hazardous and infectious medical waste should be properly managed by following the guidelines (WHO, 2020c). It is now clear that majority of the people (especially in developing countries) have a lack of knowledge regarding waste segregation and disposal issues (Rahman et al., 2020). So, government should implement extensive awareness campaign through different mass media, regarding the proper waste segregation, handling and disposal methods.
vi ***Ecological restoration and ecotourism:*** For ecological restoration, tourist spots should periodically shutdown after a certain period. Moreover, ecotourism practice should be strengthened to promote sustainable livelihoods, cultural preservation, and biodiversity conservation (Islam and Bhuiyan, 2018).
vii ***Behavioral change in daily life:*** To reduce the carbon footprint and global carbon emission, it is necessary to change the behavior in our daily life and optimum consumption or resources like; avoid processed and take locally grown food, make compost from food waste, switch off or unplug electronic devices when not used, and use a bicycle instead of a car for short(er) distances.
viii ***International cooperation:*** To meet the sustainable environmental goals and protection of global environmental resources, such as the global climate and biological diversity, combined international effort is essential (ICIMOD, 2020). Hence, responsible international authority like United Nations Environment Programme (UN Environment) should take effective role to prepare time-oriented policies, arrange international conventions, and coordination of global leaders for proper implementation.

Directly or indirectly, the pandemic is affecting human life and the global economy, which is ultimately affecting the environment and climate. It reminds us how we have neglected the environmental components and enforced human induced climate change. Moreover, the global response of COVID-19 also teaches us to work together to combat against the threat to mankind. Though the impacts of COVID-19 on the environment are short-term, united and proposed time-oriented effort can strengthen environmental sustainability and save the earth from the effects of global climate change.

## Declarations

### Author contribution statement

All authors listed have significantly contributed to the development and the writing of this article.

### Funding statement

This research did not receive any specific grant from funding agencies in the public, commercial, or not-for-profit sectors.

### Competing interest statement

The authors declare no conflict of interest.

### Additional information

No additional information is available for this paper.
